# Effect of Unfractionated Heparin Dose on Complement Activation and Selected Extracellular Vesicle Populations during Extracorporeal Membrane Oxygenation

**DOI:** 10.3390/ijms252011166

**Published:** 2024-10-17

**Authors:** Johannes Zipperle, Laurenz Vock, Gerhard Fritsch, Johannes Grillari, Marcin F. Osuchowski, Wolfgang Holnthoner, Herbert Schöchl, Rebecca Halbgebauer, Markus Huber-Lang, Nikolaus Hofmann, Vincenz Scharner, Mauro Panigada, Johannes Gratz, Giacomo Iapichino

**Affiliations:** 1 Ludwig Boltzmann Institute for Traumatology, The Research Center in Cooperation with AUVA, 1200 Vienna, Austriagerhard.fritsch@auva.at (G.F.); marcin.osuchowski@trauma.lbg.ac.at (M.F.O.); wolfgang.holnthoner@trauma.lbg.ac.at (W.H.); herbert.schoechl@medical-education.at (H.S.); 2 AUVA Trauma Center Salzburg, Department of Anaesthesiology and Intensive Care Medicine, Academic Teaching Hospital of the Paracelsus Medical University, 5010 Salzburg, Austria; 3 Austrian Cluster for Tissue Regeneration, 1200 Vienna, Austria; 4 Institute for Molecular Biotechnology, Department for Biotechnology, BOKU University, 1190 Vienna, Austria; 5 Institute of Clinical and Experimental Trauma Immunology, Ulm University Medical Center, University Hospital of Ulm, 89081 Ulm, Germanymarkus.huber-lang@uniklinik-ulm.de (M.H.-L.); 6 Department of Anaesthesia, Intensive Care Medicine and Pain Medicine, Division of General Anaesthesia and Intensive Care Medicine, Medical University of Vienna, 1090 Vienna, Austriajohannes.gratz@meduniwien.ac.at (J.G.); 7 Department of Anaesthesia, Critical Care and Emergency Medicine, Fondazione IRCCS Ca’ Granda Ospedale Maggiore Policlinico, 20122 Milan, Italy; 8 Department of Pathophysiology and Transplantation, University of Milan, 20122 Milan, Italy; 9 Department of Anesthesia and Intensive Care Medicine, IRCCS Humanitas Research Hospital, 20089 Rozzano, Italy

**Keywords:** ECMO, extracellular vesicles, anticoagulation, heparin, thrombosis, bleeding, complement, mitochondria, critical illness, biomarker

## Abstract

Extracorporeal membrane oxygenation (ECMO) provides critical support for patients with severe cardiopulmonary dysfunction. Unfractionated heparin (UFH) is used for anticoagulation to maintain circuit patency and avoid thrombotic complications, but it increases the risk of bleeding. Extracellular vesicles (EVs), nano-sized subcellular spheres with potential pro-coagulant properties, are released during cellular stress and may serve as potential targets for monitoring anticoagulation, particularly in thromboinflammation. We investigated the impact of UFH dose during ECMO therapy at the coagulation–inflammation interface level, focusing on complement activation and changes in circulating large EV (lEV) subsets. In a post hoc analysis of a multicenter randomized controlled trial comparing two anticoagulation management algorithms, we examined lEV levels and complement activation in 23 veno-venous-ECMO patients stratified by UFH dose. Blood samples were collected at different time points and grouped into three phases of ECMO therapy: initiation (day 1), mid (days 3–4), and late (days 6–7). Immunoassays detected complement activation, and flow cytometry analyzed lEV populations with an emphasis on mitochondria-carrying subsets. Patients receiving <15 IU/kg/h UFH exhibited higher levels of the complement activation product C5a and soluble terminal complement complex (sC5b-9). Lower UFH doses were linked to increased endothelial-derived lEVs, while higher doses were associated with elevated RBC-derived and mitochondria-positive lEVs. Our findings suggest the potential theranostic relevance of EV detection at the coagulation–inflammation interface. Further research is needed to standardize EV detection methods and validate these findings in larger ECMO patient cohorts.

## 1. Introduction

Extracorporeal membrane oxygenation (ECMO) is a form of extracorporeal circulation increasingly used in critically ill patients with severe cardiopulmonary dysfunction, providing continuous cardiac and/or pulmonary support as a bridge to recovery or transplantation. Systemic anticoagulation is typically necessary during ECMO to prevent excessive thrombin generation and the formation of clots on non-biological surfaces, but it remains a challenging aspect of ECMO management. The balance between bleeding and thrombosis is delicate, with both complications significantly contributing to morbidity and mortality. A recent analysis of over 7000 veno-venous (VV)ECMO patients revealed that a large portion experienced thrombotic events, bleeding events or both [1]. This highlights the challenge of carefully balancing the benefit of anticoagulation to reduce thrombotic complications against the risk of life-threatening bleeding. Unfractionated heparin (UFH), a sulfated polysaccharide that binds to antithrombin, remains the primary anticoagulant used in ECMO due to its widespread availability, affordability, and short half-life [2,3]. UFH maintains hemostatic homeostasis by promoting the binding of antithrombin to coagulation factors II and Xa [4], playing a key role in balancing coagulation and inflammation [5,6]. Despite its widespread use, current monitoring methods for UFH are not ideal. Dose adjustments are typically based on clotting times measured by standard coagulation tests such as activated partial thromboplastin time (aPTT) [7,8]. Heparin-sensitive viscoelastic assays like thromboelastography (TEG) have been investigated to complement aPTT in guiding UFH administration during VV-ECMO [9]. The complex interplay between coagulation and inflammation in ECMO patients remains an active area of research [10]. The continuous exposure of blood and the endothelium to high shear stress and extracorporeal surfaces triggers ECMO-associated inflammatory response syndrome and coagulopathy, thereby influencing patient outcomes [11]. Affected key components include leukocytes, platelets, and further elements of the coagulation and complement systems [12,13,14,15]. Complement activation, with the generation of the central anaphylatoxins C3a and C5a, leads to histamine release, increased vascular permeability, and the recruitment of immune cells [16]. C5a is also known to upregulate tissue factors (TFs) on monocytes and neutrophils and induce TF expression on endothelial cells [17,18,19]. Furthermore, complement is involved in the release of extracellular vesicles (EVs) and can be shuttled as part of their cargo, functioning remotely in an ambivalent manner [20]. EVs, as part of a nanometer-sized intercellular cargo, signaling and waste disposal system, have gained prominence as potential diagnostic biomarkers and therapeutic agents in critical illness [21]. Released from nearly all cell types, EVs carry a wide range of cargoes and facilitate intercellular transport and communication in health and disease. Variations in circulating EVs have been linked to critical illness, and specific EV signatures have been associated with ICU requirement in COVID-19 patients [22,23,24]. In COVID-19, EVs appear larger, potentially indicating the abundance of ectosomal membrane blebbing, cellular activation and/or apoptosis under critical conditions [25]. Among other means of classification, EVs are often categorized by their size, biogenesis (e.g., exosomes, ectosomes, apoptotic bodies) and/or mode of shedding (exocytosis vs. membrane blebbing) [26,27,28]. Larger EVs (lEVs), often termed microvesicles, have gained considerable interest in the intensive care setting, where patients suffer from severe diseases frequently leading to multi-organ failure, endothelial dysfunction and, ultimately, shock. The circulating lEV subsets in critical illnesses often expose pro-coagulant molecules like phosphatidylserine (PS) or TFs and have emerged as potential danger signals in various acute conditions, often disseminating a pro-inflammatory and pro-thrombotic microenvironment throughout the systemic circulation [29,30,31]. Given their role in transferring cellular components, some EV subpopulations also carry mitochondria and/or associated compounds. For instance, dysfunctional mitochondria are degraded intracellularly via lysosomal pathways; however, lysosomal inhibition increases mitochondrial secretion in large EVs, which are then captured by macrophages [32,33]. Several studies have identified mitochondrial proteins within EV cargo, but to date, their release mechanisms, functions and clinical implications still need to be elucidated [34,35,36,37]. This study is a post hoc analysis of a randomized controlled trial enrolling patients on VV-ECMO for respiratory failure. Enrolled patients randomly received two different anticoagulation protocols, resulting in significantly different anticoagulation intensities throughout the ECMO course. 

Our aim was to investigate the effect of heparin dose on the activation of pathways at the coagulation–inflammation interface, irrespective of the underlying condition and the group allocation in the original study. Specifically, we examined complement activation, circulating lEV levels and mitochondria-positive EVs in relation to anticoagulation intensity and the duration of ECMO runs. We hypothesized that a low-dose regimen would impact the physical characteristics of the blood flow within the circuit, which might eventually result in higher levels of platelet- and RBC-derived populations. We anticipated that such an occurrence would be accompanied by a higher magnitude of complement activation with a potential secondary effect on both leukocyte- and endothelial-derived lEV subsets. Furthermore, we could not exclude a direct pharmacological action of heparin on inflammatory processes, which might become apparent at higher doses of anticoagulation. Finally we hypothesized that higher levels of mitochondria-positive and monocyte/macrophage-derived vesicles might indicate the abundance of vesicular waste disposal mechanisms.

## 2. Results

Between September 2014 and November 2016, 42 participants were included in the course of the original pilot trial, 23 of whom had plasma samples available for this post hoc analysis. The basic demographics of the analyzed subgroup are given in Table 1. A detailed overview on the patient characteristics of the original cohort is published elsewhere [9].

**Figure 1:**
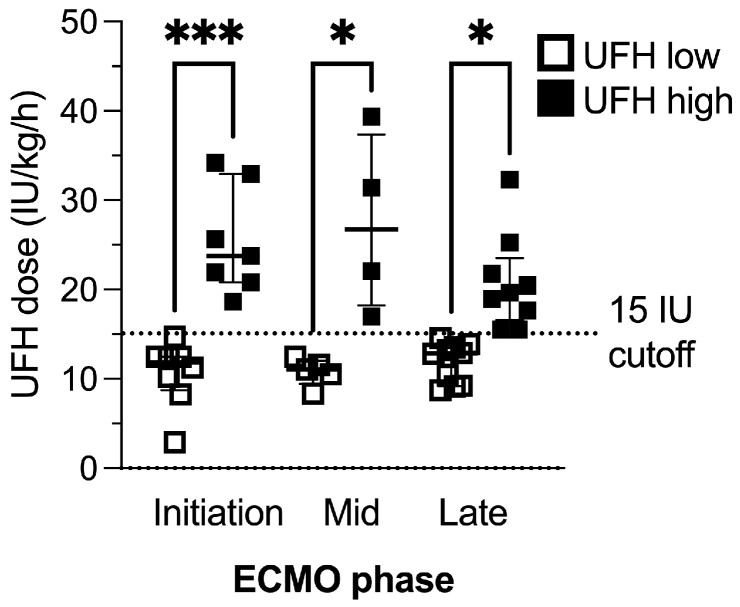
Unfractionated heparin (UFH) dose and sample size across groups at different timepoints of ECMO therapy. Sample size (N) at initiation (low: 8; high: 7), mid (low: 5; high: 4) and late (low: 10; high: 9). Data represent single measurements of each patient per day grouped by ECMO phase and UFH dose. One-way analyses of variance or Kruskal–Wallis tests were performed to compare doses across phases. Data are shown as boxes and whiskers representing the median and interquartile range. If not indicated otherwise by lines and asterisks, differences in groupwise comparisons are non-significant. * = *p* < 0.05; *** *p* < 0.001.

Based on a cutoff dose of 15 IU/kg/h, 23 sampling points were assigned to the UFH low group (Median IU 11.57; IQR 9.21–12.88), whereas 20 sampling points were included in the UFH high group (Median IU 21.84; IQR 18.71–29.97). A detailed overview of UFH dose and sample size throughout the defined ECMO phases is given in Figure 1. 

### 2.1. Complement Activation during ECMO Is More Pronounced at Lower Dose Anticoagulation

**Figure 2:**
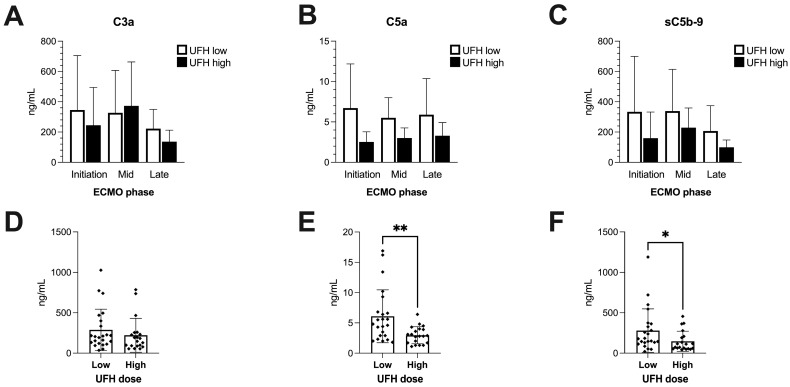
Complement activation throughout ECMO treatment and in comparison to UFH dose regimens. Activated complement pathway components C3, C5 and sC5b-9 at absolute concentrations in plasma of ECMO patients at different timepoints of ECMO therapy (**A**–**C**). One-way analyses of variance or Kruskal–Wallis tests were performed to compare phases and doses. Data are shown as columns and whiskers representing mean and SD. Comparisons of UFH doses in all patients and at all time points undergoing ECMO (**D**–**F**). Data are plotted as columns and whiskers representing mean and SD. A Student *t*-test or Mann–Whitney test was performed to compare groups. Sample size (N) in ECMO phases: initiation (low: 8; high: 7), mid (low: 5; high: 4), and late (low: 10; high: 9). Sample size (N) in summary measures: UFH dose low: 23; UFH dose high: 20. If not otherwise indicated by lines and asterisks, differences in groupwise comparisons were non-significant. * = *p* < 0.05; ** = *p* < 0.01.

The levels of the central anaphylatoxins C3a and C5a, as well as the soluble terminal complement complex (sC5b-9), did not show differences between UFH dose groups for any of the predefined time phases of ECMO treatment (Figure 2A–C). However, summary measures for all timepoints revealed significantly higher levels of C5a and sC5b-9 in the UHF low group (Figure 2E,F), but not for the complement component C3a (Figure 2D). 

### 2.2. Anticoagulation Dose Affects Circulating lEV Populations

**Figure 3:**
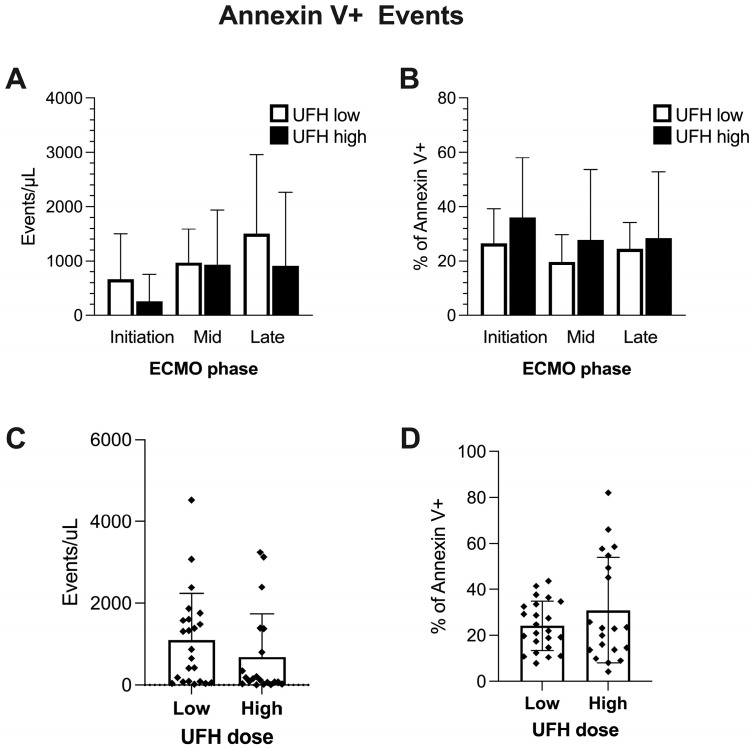
Annexin V+ events throughout the ECMO period and in comparison to UFH dose regimens, as analyzed by flow cytometry. Absolute and relative levels of Annexin V+ events in plasma of ECMO patients at different stages of ECMO therapy (**A**,**B**). One-way analyses of variance or Kruskal–Wallis tests were performed to compare phases and doses. Data are shown as columns and whiskers representing mean and SD. Effect of UFH dose on absolute and relative counts of Annexin V+ events in all patients and at all time points undergoing ECMO (**C**,**D**). Data are plotted as columns and whiskers representing mean and SD. A Student *t*-test or Mann–Whitney test was performed to compare groups. Sample size (N) in ECMO phases: initiation (low: 8; high: 7), mid (low: 5; high: 4), and late (low: 10; high: 9). Sample size (N) in summary measures: UFH dose low: 23; UFH dose high: 20. If not indicated otherwise by lines and asterisks, differences in groupwise comparisons were non-significant.

When investigating the levels of lEVs based on the discriminating marker Annexin V on circulating populations, we found no differences between UFH low and UFH high (Figure 3A,B). The summary measures revealed a trend (*p* = 0.1) towards higher absolute levels of Annexin V+ lEVs in the low-dose group (Figure 3C), although this did not reach statistical significance (Figure 3B,D). 

### 2.3. Cellular Origin and Quantification of Extracellular Vesicles during ECMO Phases

**Figure 4:**
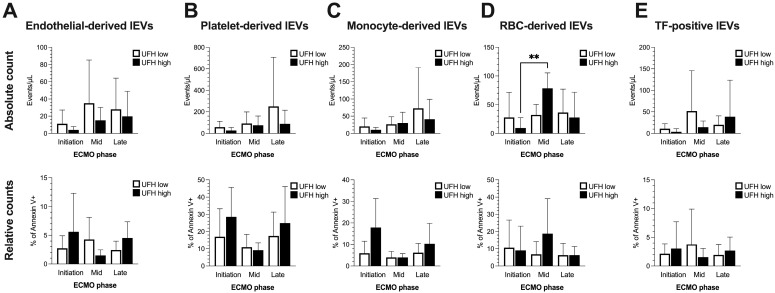
Absolute (events/μL, upper row) and relative counts (% of Annexin V positive events, lower row) of lEV populations of different cellular origins throughout different phases of ECMO therapy (**A**–**E**). One-way analyses of variance or Kruskal–Wallis tests were performed to compare phases and doses. Data are shown as columns and whiskers representing mean and SD. Sample size (N) in ECMO phases: initiation (low: 8; high: 7), mid (low: 5; high: 4), and late (low: 10; high: 9). If not indicated otherwise by lines and asterisks, differences in groupwise comparisons were non-significant. RBCs (red blood cells); TF (tissue factor). ** = *p* < 0.01.

Based on Annexin V’s positivity as a discriminator, several lineage-specific antigen combinations were used to identify the cellular origin of lEVs in the blood of patients undergoing ECMO (Figure 4). We identified vesicles from endothelial cells (AnnV+, CD31+, CD42b-), platelets (AnnV+, CD31+, CD42b+), monocytes (AnnV+, CD14+) and RBCs (AnnV+, CD235a+), as well as TF-positive IEVs (AnnV+, CD142+; irrespective of cellular origin). The absolute quantification of lEVs revealed an increase in RBC-derived lEVs in the higher-UFH-dose group that was not detectable at a lower dose (Figure 4D, upper row). None of the other assessed lEV populations showed significant changes in absolute count throughout ECMO (Figure 4A–C,E, upper row). The increase in RBC-derived lEVs was not paralleled by a general shift of populations towards a larger RBC percentage in Annexin V+ events (Figure 4D, lower row). Similarly, none of the analyzed lEVs fractions were significantly altered over time with regard to relative contribution to the overall Annexin V+ population. This was irrespective of the UFH dose (Figure 4A–E, lower row). 

**Figure 5:**
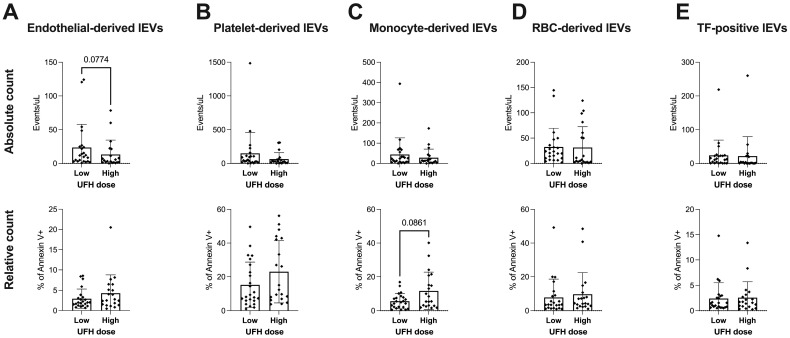
Absolute (events/μL, upper row) and relative counts (% of Annexin V positive events, lower row) of lEV populations of different cellular origins depending on anticoagulation regimens throughout ECMO therapy (**A**–**E**). Student *t*-tests or Mann–Whitney tests were performed to compare doses. Data are shown as columns and whiskers representing mean and SD. *p*-values below 0.1 are given to indicate trends. Sample size (N) in summary measures: UFH dose low: 23; UFH dose high: 20. If not indicated otherwise by lines and asterisks, differences in groupwise comparisons were non-significant.

Summary measures based on UFH dose revealed no group differences in the absolute or relative counts of lEVs of different cellular origins (Figure 5A–E). Although this did not reach statistical significance, we observed a trend towards higher absolute levels of endothelial-derived lEVs in the lower dose and a higher relative count of monocyte/macrophage-derived lEVs in the higher UFH group (Figure 5A,C).

### 2.4. Higher UFH Dose during ECMO Resulted in Elevated Levels of lEVs Carrying Mitochondria

**Figure 6:**
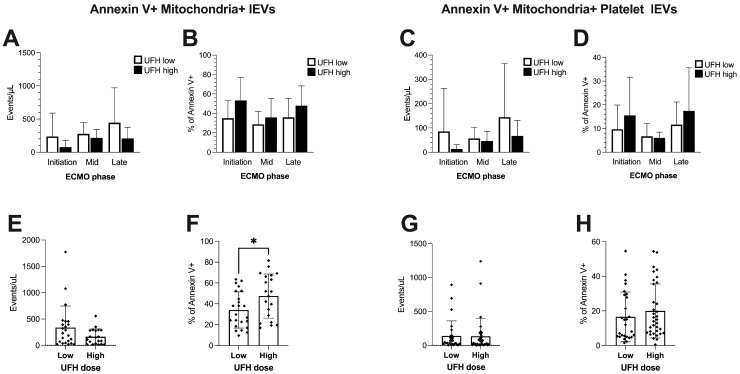
Absolute (events/μL) and relative count (% of Annexin V-positive events) of Mitochondria+ lEV populations throughout ECMO phases and in comparison to UFH dose regimens. Annexin V+- and MitoTracker DeepRed double-positive events in plasma of ECMO patients at different stages of ECMO therapy (**A**,**B**) and grouped by UFH dose (**E**,**F**). Annexin V+-, MitoTracker- DeepRed- and platelet CD42b-positive events in plasma of ECMO patients at different stages of ECMO therapy (**C**,**D**) and grouped by UFH dose (**G**,**H**). One-way analyses of variance or Kruskal–Wallis tests were performed to compare selected phases and doses. Data are shown as columns and whiskers representing mean and SD. A Student *t*-test or Mann–Whitney test was performed to compare groups based on UFH dose. Sample size (N) in ECMO phases: initiation (low: 8; high: 7), mid (low: 5; high: 4), and late (low: 10; high: 9). Sample size (N) in summary measures: UFH dose low: 23; UFH dose high: 20. If not indicated otherwise by lines and asterisks, differences in groupwise comparisons were non-significant. * = *p* < 0.05.

From the entirety of circulating Annexin V-positive populations, absolute and relative lEVs with a mitochondrial fluorophore signal were identified (Figure 6A,B,E,F). In the high-dose UFH group, a significantly higher relative portion of lEVs was positive for mitochondria (Figure 6F), but there was no difference in the absolute numbers of this subpopulation (Figure 6E). Furthermore, mitochondria-positive lEV populations were not significantly altered during particular ECMO phases (Figure 6A,B). Further identification of mitochondria-positive lEV subsets with an emphasis on platelet CD42b-positive events revealed no changes in relative or absolute counts in this population over time, irrespective of UFH dose (Figure 6C,D). The same held true in the pooled data sets (Figure 6G,H). 

## 3. Discussion

Our study focusing on (i) complement activation, (ii) Annexin V+ lEVs from different cellular origin and (iii) the mitochondrial content of this subset revealed that the UFH maintenance dose during ECMO affected quantitative and temporal changes in the investigated entities. 

As expected, ECMO led to an overall activation of complement, which was likely due to the large artificial surfaces of the ECMO circulation known to induce thromboinflammation [38]. This is substantiated by previous experimental studies, where the coating of the ECMO biomaterial surfaces with heparin resulted in a decrease in complement activation [39]. In the current study, patients receiving <15 IU/kg/h of heparin displayed a higher magnitude of complement activation with regard to C5a and the formation of the terminal complement complex sC5b-9. They also showed a trend towards higher absolute levels of Annexin V+ lEVs, particularly from an apparent endothelial origin. Conversely, patients who were administered a higher dose of UFH revealed an increase in the absolute quantity of erythrocyte (RBC)-derived lEVs after a few days on ECMO, which was absent in the lower-dose group. This phenomenon might be attributed to occlusive events within the circuit, especially within the oxygenator, causing increased RBC stress, hemolysis and EV shedding. Under such circumstances, the attending physician might react to signs of circuit thrombosis (increasing transmembrane pressure, impaired artificial gas exchange, increasing d-dimers or consumption coagulopathy) by advancing anticoagulation through an increase in administered UFH. It must also be mentioned that despite the fact that transfusion protocols were adjusted to maintain a uniform target hemoglobin concentration of 10 g/dL in all patients, individual fluctuations in RBC- and platelet-derived lEVs might also be attributed to the administration of blood products containing this particular subset. At a higher UFH dose, a larger portion of the circulating Annexin V+ subset also appeared to carry mitochondria, even though the specific cellular origin of this population could not be determined. 

The optimal dose of anticoagulants (such as UFH) and respective monitoring strategies during ECMO are still a matter of debate and largely depend on defined target values in laboratory assays (e.g., anti Xa activity, prolongation of clotting times in aPTT, ACT, clot initiation time at thromboelastometry/thromboelastography, etc.). In a retrospective single-center study of patients on VV-ECMO, a lower incidence of major bleeding and bleeding-related deaths was observed in those receiving low-dose UFH compared to those on therapeutic-dose UFH, without a significant difference in the need for oxygenator changes or major thrombotic events [40]. Conversely, no significant differences in survival rates, bleeding or thromboembolic events and transfusion requirements were found in a retrospective study comparing low-dose and full-dose UFH strategies in VV-ECMO [41]. A further study enrolling VV-ECMO patients reported no differences in rates of thrombosis, bleeding complications or mortality between low-dose and therapeutic-dose UFH groups, although the small sample size was a limiting factor [42]. A pilot RCT with VV-ECMO patients showed no differences in oxygenator changes, transfusions and bleeding complications between standard-dose UFH and a non-titrating weight-based UFH infusion [43]. Other clinical trials (RATE-trial, NCT04536272; CASUAL-ECMO trial NCT06442267) are still ongoing and aim to further evaluate different anticoagulation strategies in ECMO patients to provide more definitive evidence on optimal anticoagulation targets. 

The uncertainty with regard to optimal anticoagulation is also attributed to the multitude of confounders that affect the incidence of complications like circuit thrombosis or bleeding. In addition to the heterogeneity of subjects and conditions that require ECMO, there are technical variations in material coatings, pump systems and fluids that affect shear forces and the coagulant state beyond pathophysiological or pharmacological factors. Certain materials appear to promote thromboinflammation, which is triggered by the interaction of biomaterials, cell surfaces and plasmatic blood components and can be mitigated by anti-inflammatory modifications like specific complement inhibitors [14]. This is in line with studies on VA-ECMO in cardiogenic shock that reported an increase in circulating immature neutrophils, decreased C5a receptor expression (generally reflecting higher quantities of circulating C5a), lymphocyte dysfunction, the expansion of myeloid-derived suppressor cells and elevated levels of both pro-inflammatory and anti-inflammatory cytokines [12]. While artificial biomaterials are generally less immunologically active than transplants or tissue-derived materials, they often have pro-coagulant properties and are capable of directly activating complement pathways [38]. This activation can induce a local and systemic inflammatory response, mediated by complement components that act as ligands to immune cells and the endothelium [44]. In a similar fashion, lEVs that are released from these activated and/or apoptotic cells also function as endocrine conveyors of a pro-inflammatory or pro-thrombotic milieu throughout the circulation. During activation and apoptosis, cells expose the intracellular phospholipid phosphatidylserine (PS) via a flip mechanism. It is therefore used as a target for the detection of cellular decay and is also abundant on subsets of EVs upon release from the surface membrane of affected cells. PS also acts as a catalyst for the formation of coagulation factor complexes and together with TF contributes to the pro-thrombotic potential of certain EV subsets [30]. Fluorophore-labeled Annexin V binds to PS in a reversible, Ca^++^-dependent manner and together with other reagents has been established as marker for this heterogenous EV subset in flow cytometry. Using low-speed (16,100× *g*) centrifugation for the enrichment of potential EVs from plasma and adding Annexin V as a discriminator, we were able to focus on the larger subset of EVs, which is released from cells undergoing activation and/or apoptosis. In accordance with our hypothesis, this lEV subset was likely affected by both the underlying condition and the invasive nature of ECMO therapy. Based on our findings that did not indicate significant changes in the selected cellular subsets over time (except for the RBC fraction), we suspected the general occurrence of higher levels of lEVs and cellular debris during ECMO. Such circumstances may result in assay saturation and an adverse signal-to-noise ratio with a potential masking effect on anticoagulation or other EV-associated phenomena. When analyzing EV populations, both total concentrations (events/µL) and relative portions (% Annexin V+) can be reported. This dual approach allows for a comprehensive view, as relative portions may remain constant when all subsets increase equally in absolute terms or may appear smaller if one subset disproportionately increases. By providing both absolute and relative data, we aimed to offer a complete understanding of the changes in EV subsets, regardless of statistical significance. 

In our attempt to focus on potentially detrimental mechanisms, we further investigated the abundance of mitochondrial content in the two different UFH dose groups. The significance of mitochondria in the composition and biological activity of EVs has formerly been demonstrated in acute and chronic inflammation [36]. Activated monocytic cells release free and EV-encapsulated mitochondria that carry TNFα and interferonogenic mitochondrial RNA, which can induce inflammatory pathways in target cells. Therefore, it has been proposed to consider mitochondria and their release mechanisms as therapeutic targets in certain pro-inflammatory conditions. However, since EV release often occurs as a waste disposal mechanism, not all mitochondria-positive lEVs may be considered danger signals. In cardiac homeostasis, the release of these organelles in lEVs constitutes a mechanism of mitochondrial quality control when lysosomal function is compromised. In aged mice and Danon disease patients, mitochondria are eliminated via the endosomal pathway, with secreted EVs being captured by macrophages without further activating these cells [32]. Our data indicating elevated levels of mitochondria-positive lEVs and a simultaneous trend towards the abundance of monocyte/macrophage-derived lEVs in the higher-dose UFH group substantiate this hypothesis, but it appears questionable to assign these observations to either a detrimental or regenerative phenotype. 

## 4. Materials and Methods

### 4.1. Study Design and Eligible Subjects

The current post hoc analysis was part of a larger study aimed at evaluating thromboelastography-guided anticoagulation management during extracorporeal membrane oxygenation [9]. The original multicenter, prospective, and randomized pilot trial was conducted at two Italian ECMO referral hospitals: (1) Fondazione IRCCS Ca’ Granda—Ospedale Maggiore Policlinico in Milan and (2) ISMETT Istituto Mediterraneo per i Trapianti e Terapie ad Alta Specializzazione in Palermo. The trial was registered at ClinicalTrials.gov (identifier: NCT02271126). The study protocol received approval from the ethics committees of both centers (Milano Area 2; Decision no. 452 from 14 March 2017, Decision no. 556 from 30 March 2017), and informed consent was obtained in accordance with Italian regulations. This study included patients who required VV-ECMO for acute hypoxemic respiratory failure of different causes. In order to follow a uniform transfusion protocol, all patients were maintained at a hemoglobin target concentration of 10 g/dL throughout the study period. Exclusion criteria were veno-arterial ECMO, an age under 18 years old, heparin-induced thrombocytopenia (HIT), a platelet count below 30,000/mm^3^, and acute respiratory failure post-lung transplant. The study period concluded upon ECMO disconnection, lung transplant (for bridge to transplant cases), patient death, or the onset of HIT. The entire study protocol, inclusion flow chart and patient characteristics are published in detail elsewhere [9]. Upon completion of this study, additional analyses on the remainder of samples were performed at the Ludwig Boltzmann Institute for Traumatology; a research center working in cooperation with AUVA in Vienna, Austria; and the Institute of Clinical and Experimental Trauma Immunology, Ulm University Medical Center, Ulm, Germany, where the data were analyzed and interpreted in cooperation with the Department of Anaesthesia, Intensive Care Medicine and Pain Medicine, the Medical University of Vienna, Austria. The available plasma samples were analyzed and grouped based on the UFH maintenance dose in international units (IU)/kg body weight per hour. The cutoff level for UFH was set at 15 IU/kg/h considering the median dose in both groups of the original study: aPTT 15.7 (10.9–21.3) vs. TEG 11.7 (9.5–15.3). Patients were allocated to one of two groups, <15 IU/kg/h (UFH low) and >15 IU/kg/h (UFH high), according to the maintenance dose of UFH that the patient was receiving at the moment of sampling. Due to expected changes in lEV populations over time, group comparisons were structured around predefined ECMO phases, also accounting for plasma sample availability, as follows: initiation (D1), mid (D3–4) and late (D6–7). This study design enabled the reassignment of single patients and sampling points to either of the groups in case the UFH dose was lowered or increased. Summary measures included all sampling points and patients per UFH dose group. Duplicate measurements per phase and dose group, as well as data without UFH administration (prior to ECMO and after removal), were not considered for analysis. The overall numerosity at each time point varied according to the availability of samples; therefore, some patients were not represented at every time point.

### 4.2. Sample Collection

During the original study, blood samples were collected at both recruiting centers from central venous lines already placed into tubes containing 3.2% trisodium citrate as an anticoagulant. Within two hours of collection, the blood was centrifuged at 2000× *g* for 15 min to obtain platelet-poor plasma. This plasma was aliquoted into Eppendorf tubes, frozen and stored at −80 °C until further processing. For shipment, one vial of these samples from different relevant time points for each patient was selected and transported by an international carrier in a monitored dry ice container to prevent thawing. 

### 4.3. Flow Cytometry of lEV Populations to Determine Cellular Origin

Extracellular vesicle enrichment was performed through differential centrifugation designed to enrich larger EVs without the necessity of ultracentrifugation, chromatography or other methods of isolation. Enrichment and analytical procedures were optimized to be carried out with a commercial tabletop centrifuge (Eppendorf 5415 R, fixed angle rotor, Hamburg, Germany) under moderate resource settings. Initially, the plasma samples were centrifuged at 16,100× *g* for 30 min at 4 °C. Under these conditions, larger EV fractions, cellular debris and aggregates were spun down, whereas smaller EVs and non-cellular compounds were presumed to be retained in the supernatant. Post-centrifugation, the supernatant was discarded and the pellet was carefully resuspended in 200 μL PBS-BSA. The resuspended pellet was then split into two 100 μL aliquots for the two optimized flow cytometry panels. One of the aliquots was co-stained with a range of specific antibodies. These included a labeled monoclonal antibody targeting platelet endothelial cell adhesion molecule-1 (PECAM-1, eBioscience Anti-Human CD31– eBioscience™, PerCP-eFluor™710, San Diego, CA, USA), a monoclonal antibody against platelet glycoprotein Ib alpha chain (CD42b, GPIb, FITC, eBioscience™, San Diego, CA, USA), an antibody against the human glycophorin CD235a (APC, eBioscience™, San Diego, CA, USA), an antibody against the monocyte/macrophage TLR co-receptor 2 (CD14, clone 61D3, APC- eFluor™780, eBioscience™, San Diego, CA, USA) and an antibody for tissue factor (CD142, clone HTF-1, PE, eBioscience™, San Diego, CA, USA). Each antibody solution was spun down at 16,000× *g* for 10 min prior to staining. 

The samples were incubated on ice in the dark for 30 min and then washed with 1mL of sterile-filtered PBS-BSA (1%), followed by another centrifugation step at 16,100× *g* for 30 min at 4 °C to spin down potential lEVs. All further steps were performed equally in both panel aliquots. 

### 4.4. Detection of lEVs Carrying Mitochondria

For the detection of mitochondrial cargo, the second aliquot was stained with 5 μL of Mitotracker DeepRed, a non-toxic fluorescent chemical probe with a thiol-reactive chloromethyl-moiety that is accumulated in mitochondria of both fixed and unfixed cells (MitoTracker™ Deep Red FM Dye for flow cytometry, 150 μM, cat. Nr. M46753, Invitrogen, San Diego, CA, USA). Together with 3 μL of the platelet CD42b antibody used in the cellular origin panel, samples were incubated at 37 °C for 30 min and then washed with 1mL of sterile-filtered Phosphate-Buffered Saline with 1% Bovine Serum Albumin (PBS-BSA, 1%; Sigma Aldrich, St. Louis, MO, USA), followed by another centrifugation step at 16,100× *g* for 30 min at 4 °C to pellet potential lEVs. In both panel aliquots, supernatants were discarded and the pellet was resuspended in 300 μL of 1× Annexin V binding buffer (Annexin V binding buffer solution, 10×, Invitrogen eBioscience™, San Diego, CA, USA) to which 5 μL of fluorophore-labeled Annexin V staining reagent (Annexin V-PE-Cy7, Invitrogen eBioscience™, San Diego, CA, USA) was added. Samples were briefly incubated on ice for 15 min to allow Annexin V binding before flow cytometric analysis. Flow cytometric analysis was conducted using a CytoFLEX flow cytometer (Beckman Coulter, Brea, CA, USA) equipped with CytExpert software version 1.2 from Beckman Coulter. Daily quality control was performed using CytoFLEX Daily QC Fluorospheres (Beckman Coulter) to ensure instrument accuracy on a daily basis. Single-stained lEV samples and fluorescence-minus-one (FMO) controls were used to establish compensation matrices and address spectral overlap due to the use of multiple fluorophores. Annexin V-positivity was determined with binding buffer vs. PBS experiments and used as a discriminator for subsequent detection and gating strategies. Due to conservative gating, none of the Annexin V measurements reached 100% of Annexin V+ events.

### 4.5. Complement ELISA 

Complement factors in plasma were measured using commercially available kits according to the manufacturers’ instructions. C3a was determined using the MicroVue™ C3a Plus EIA (QuidelOrtho Corp., San Diego, CA, USA), C5a was determined employing the C5a ELISA (DRG Diagnostics, Marburg, Germany), and sC5b-9 (formerly known as membrane attack complex, MAC) was assessed using the BD OptEIA™ Human C5b-9 ELISA Set (BD Biosciences, San Diego, CA, USA).

### 4.6. Statistical Analysis

Data were collected and managed in Microsoft Excel 2019, and statistical analyses were performed using GraphPad Prism 8.0. The normality of data distribution was evaluated using a Kolmogorov–Smirnov test. Depending on the distribution, either one-way ANOVAs or Kruskal–Wallis tests were used for comparisons. In accordance with our hypotheses, inter-group comparisons were performed to study the effect of UFH dose at all ECMO stages, whereas groupwise comparisons between phases were carried out to study temporal changes in lEV quantity and composition. For comparison of pooled measurements based on UFH dose, Student *t*-tests or Mann–Whitney tests were performed depending on normality. If not indicated otherwise, all data were displayed as mean ± SD. Categorical variables were displayed in absolute numbers or percentage of total; continuous variables were displayed as mean ± SD. *p* values ≤ 0.1 were described as trends or tendencies and were pointed out in the respective graphs. The level of statistical significance was set at *p* < 0.05.

### 4.7. Limitations

The small sample size and retrospective nature of this study limit the generalizability of our results. Additionally, enrolled patients have different etiologies of respiratory failure, leading to possible heterogeneity in the baseline coagulation and inflammatory status. Apart from that, the multifactorial conditions of the critically ill ECMO patients in our study, including severe infections and organ failure, may influence some of our observations, including complement activation and IEV formation. Additionally, despite efforts to uniformly stratify by UFH dose, other confounding factors cannot be fully excluded in this post hoc analysis. In particular, these limitations do not allow the direct translation of our findings into clinical practice. Additionally, the processes used for EV enrichment and detection are not standardized, leading to significant analytical variability and susceptibility to errors. Although our experimental approach offers valuable insights into the abundance of specific EV subtypes, it does not capture the full spectrum of circulating EV populations and does not comply with all recommendations for the characterization of EVs according to the 2023 MISEV guidelines [28]. In particular, the discrimination of lEVs based on Annexin V positivity neglects the vast array of subpopulations that are not enriched or detected by the employed technologies. Another limitation of our study is the variability in pharmacokinetics of unfractionated heparin (UFH), with individual alterations in heparin response. Furthermore, as aPTT has known drawbacks in critically ill patients, particularly its dependence on FVIII and FXII levels, future studies should consider the use of anti-Xa levels for more accurate monitoring of heparin therapy.

## 5. Conclusions

We showed that UFH dose impacts the concentrations and varieties of circulating lEVs during ECMO. Lower UFH doses correlated with an increased complement activation and rise in Annexin V+/endothelial-derived lEVs, whereas higher doses were associated with an increased number of RBC-derived and mitochondria-positive lEVs. Our findings shed further light on the complex coagulation–inflammation interplay during ECMO therapy. Additional research is necessary to standardize EV detection techniques, validate our observations in larger cohorts and evaluate their possible future theragnostic potential for clinical practice.

## Figures and Tables

**Table 1:** The patient characteristics of the cohort enrolled in the post hoc analysis. Categorical variables are given in absolute numbers or as a percentage of the total; continuous variables are displayed as mean ± SD. ARDS (Acute Respiratory Distress Syndrome); TEG (Thromboelastography); aPTT (activated Partial Thromboplastin Time);.

**Number of enrolled patients**	23
Age (years)	45 ± 14
Weight (kg)	73 ± 25
Male sex (%)	52%
Number of ECMO days	12 ± 7
Number of patients undergoing at least one ECMO circuit change (%)	8 (35%)
**Diagnosis (% of total)**	
ARDS	15 (65%)
Bridge to lung transplantation	7 (31%)
Status asthmaticus	1 (4%)
**Group allocation**	
TEG	48%
aPTT	52%

## Data Availability

All data analyzed in the current study are available from the corresponding author upon reasonable request.
